# Human N-acetyltransferase 2 (*NAT2*) gene variability in Brazilian populations from different geographical areas

**DOI:** 10.3389/fphar.2023.1278720

**Published:** 2023-11-15

**Authors:** Márcia Quinhones P. Lopes, Raquel Lima F. Teixeira, Pedro Hernan Cabello, José Augusto C. Nery, Anna Maria Sales, Edilbert Pellegrini Nahn J. R., Marilda Vieira Moreira, Ewalda Von Rosen Stahlke, Lia Gonçalves Possuelo, Maria Lucia R. Rossetti, Marcelo F. Rabahi, Luciana F. M. Silva, Patrícia Almeida Leme, William John Woods, Mauricio Lisboa Nobre, Maria Leide Wan-Del-Rey de Oliveira, Kazuê Narahashi, Milde Cavalcanti, Philip Noel Suffys, Sotiria Boukouvala, Maria Eugênia N. Gallo, Adalberto Rezende Santos

**Affiliations:** ^1^ Laboratory of Molecular Biology Applied to Mycobacteria, Oswaldo Cruz Institute, Oswaldo Cruz Foundation, Rio de Janeiro, RJ, Brazil; ^2^ Laboratory of Human Genetics, Oswaldo Cruz Institute, Oswaldo Cruz Foundation, Rio de Janeiro, RJ, Brazil; ^3^ Leprosy Laboratory, Souza Araújo Outpatient Clinic, Oswaldo Cruz Institute, Oswaldo Cruz Foundation, Rio de Janeiro, RJ, Brazil; ^4^ Leprosy Outpatient Clinic, Northern Fluminense State University, Campos dos Goytacazes, RJ, Brazil; ^5^ Holy House of Mercy of Espírito Santo, Vitória, ES, Brazil; ^6^ Metropolitan Regional Specialty Center, Curitiba, PR, Brazil; ^7^ Department of Molecular Biology and Biotechnology, IB and Biotechnology Center, Federal University of Rio Grande do Sul, Porto Alegre, RS, Brazil; ^8^ Anuar Auad Infectious Disease Reference Hospital, Goiania, GO, Brazil; ^9^ Municipal Secretary of Health of Palmas, Palmas, TO, Brazil; ^10^ Municipal Secretary of Health of Gurupi, Gurupi, TO, Brazil; ^11^ Coordination of Sanitary Dermatology, Rio Branco, AC, Brazil; ^12^ Giselda Trigueiro Hospital, Natal, RN, Brazil; ^13^ Sector of Dermatology, Federal University of Rio de Janeiro, Rio de Janeiro, RJ, Brazil; ^14^ Polyclinic Oswaldo Cruz, Porto Velho, RO, Brazil; ^15^ Polyclinic Clementino Fraga, Recife, PE, Brazil; ^16^ Laboratory of Molecular Genetics and Pharmacogenomics - Toxicogenomics, Department of Molecular Biology and Genetics, Democritus University of Thrace, Alexandroupolis, Greece

**Keywords:** human NAT2, N-acetyltransferase 2, polymorphisms, admixed population, sequencing

## Abstract

**Introduction:** Several polymorphisms altering the *NAT2* activity have already been identified. The geographical distribution of *NAT2* variants has been extensively studied and has been demonstrated to vary significantly among different ethnic population. Here, we describe the genetic variability of human N-acetyltransferase 2 (*NAT2*) gene and the predominant genotype-deduced acetylation profiles of Brazilians.

**Methods:** A total of 964 individuals, from five geographical different regions, were genotyped for *NAT2* by sequencing the entire coding exon.

**Results:** Twenty-three previously described NAT2 single nucleotide polymorphisms (SNPs) were identified, including the seven most common ones globally (c.191G>A, c.282C>T, c.341T>C, c.481C>T, c.590G>A, c.803A>G and c.857G>A). The main allelic groups were NAT2*5 (36%) and NAT2*6 (18.2%), followed to the reference allele NAT2*4 (20.4%). Combined into genotypes, the most prevalent allelic groups were NAT2*5/*5 (14.6%), NAT2*5/*6 (11.9%) and NAT2*6/*6 (6.2%). The genotype deduced *NAT2* slow acetylation phenotype was predominant but showed significant variability between geographical regions. The prevalence of slow acetylation phenotype was higher in the Northeast, North and Midwest (51.3%, 45.5% and 41.5%, respectively) of the country. In the Southeast, the intermediate acetylation phenotype was the most prevalent (40.3%) and, in the South, the prevalence of rapid acetylation phenotype was significantly higher (36.7%), when compared to other Brazilian states (*p* < 0.0001). Comparison of the predicted acetylation profile among regions showed homogeneity among the North and Northeast but was significantly different when compared to the Southeast (*p* = 0.0396). The Southern region was significantly different from all other regions (*p* < 0.0001).

**Discussion:** This study contributes not only to current knowledge of the *NAT2* population genetic diversity in different geographical regions of Brazil, but also to the reconstruction of a more accurate phenotypic picture of *NAT2* acetylator profiles in those regions.

## 1 Introduction

Pharmacogenetics refers to the study of genetic differences in metabolic pathways that can affect individual response to drugs, either in terms of therapeutic efficacy or risk for adverse reactions. N-acetylation is a primary detoxification route for several drugs and carcinogens (Hein, 2002). N-acetyltransferase 1 and 2 genes (*NAT1* and *NAT2*), encoding for phase II xenobiotic-metabolizing NAT1 and NAT2 enzymes, represent one of the very first landmark examples of how genetic variation among individuals and across populations may affect drug safety (Evans, 1989). It was shown, in the early days of pharmacogenetics, that the bioavailability of the tuberculostatic drug isoniazid was dependent on polymorphic NAT2 mediated N-acetylation, where a clearly bimodal population distribution of plasma elimination half-life distinguished individuals as phenotypically “rapid” or “slow” acetylators (Evans et al., 1960). In later years, a trimodal population distribution pattern was recognized as representing the NAT2 phenotype more accurately with slow, intermediate and rapid acetylators bearing two, one or no defective *NAT2* alleles, respectively (Parkin et al., 1997; Chen et al., 2006; Zang et al., 2007; Walraven et al., 2008). The “slow” NAT2 phenotype has since been investigated in the literature as a biochemically co-dominant, but clinically recessive, autosomal Mendelian trait and the genotype is considered as highly predictive of the phenotype (Boukouvala and Fakis, 2005; Sim et al., 2012).

In addition to isoniazid (INH), hydralazine, dapsone, procainamide, amifampridine, various sulphonamides and other clinically important drugs (Weber and Hein, 1985, also see the FDA Table of Pharmacogenomic Biomarkers in Drug Labeling, Food and Drug Administration, 2022) are dependent on metabolic detoxification via the N-acetylation pathway, with poor metabolizers demonstrating higher risk for adverse reactions (Palamanda et al., 1995; Ohno et al., 2000; Furet et al., 2002; Magalon et al., 2008; McDonagh et al., 2014). The influence of *NAT2* genetic variability to the acetylation phenotype and, consequently, to the therapeutic outcomes of those drugs has been demonstrated for several disease models, including for tuberculosis and hypertension among others (Possuelo et al., 2008; Teixeira et al., 2011; Spinasse et al., 2014). Moreover, *NAT2* variability has been investigated as a possible risk factor for xenobiotic-related susceptibility to Alzheimer’s disease, schizophrenia, diabetes, cataract, and parkinsonism (Bialecka et al., 2002; Agúndez et al., 2008; Luan et al., 2017), while it is also linked to urinary bladder and colorectal cancers (García-Closas et al., 2005; An et al., 2015).

*NAT2* is a highly polymorphic gene on chromosome 8p22, with the majority of single nucleotide polymorphisms (SNPs) located within its 873 bp intronless coding region (McDonagh et al., 2014, also NAT website, http://nat.mbg.duth.gr/). Database of arylamine N-acetyltransferase (2016) Currently, more than 30 SNPs have been identified, combined to form 108 different alleles that can be classified into 19 distinct haplotypic groups. This classification is based on the presence of specific “signature” SNPs relative to the NAT2*4 reference allele, which is the most common functional (or “rapid”) allele across global populations (Vatsis et al., 1995; Hein et al., 2008; Boukouvala, 2018). Seven SNPs are particularly frequent in all populations; four of those cause amino acid changes which lead to a significant decrease in acetylation activity of the NAT2 isoenzyme and are associated with the slow acetylation phenotype. Those are rs1801280 (c.341T>C, p.Ile114Thr; signature SNP for the NAT2*5 allelic group), rs1799930 (c.590G>A, p.Arg197Gln; signature SNP for the NAT2*6 allelic group), rs1799931 (c.857G>A, p.Gly286Glu; signature SNP for the NAT2*7 allelic group) and rs1801279 (c.191G>A, p.Arg64Gln; signature SNP for the NAT2*14 allelic group). The remaining three common SNPs have no effect on the phenotype. Those are rs1041983 (c.282C>T, p.Tyr94=; signature SNP for the NAT2*13 allelic group), rs1799929 (c.481C>T, p.Leu161=; signature SNP for the NAT2*11 allelic group) and rs1208 (c.803A>G, p.Lys268Arg; signature SNP for the NAT2*12 allelic group) (Fretland et al., 2001; Hein, 2006; Zang et al., 2007; Hein and Millner, 2021), and are encountered in numerous haplotypes across different allelic groups associated with both rapid and slow acetylation. Except for the NAT2*4 reference allele, all other alleles carry various combinations of up to six different SNPs in the same haplotype. The diplotypic combinations of different “rapid” or “slow” *NAT2* alleles result in variable genotypes that can be used to accurately determine the phenotype as rapid, intermediate or poor metabolizer of the investigated drug.

Because of its medical interest, the geographical population distribution of *NAT2* variants has been extensively studied and has been demonstrated to vary significantly among different ethnic backgrounds (Patin et al., 2006a; Teixeira et al., 2007; Sabbagh et al., 2008; Eny et al., 2014; Tiis et al., 2020; Hein and Millner, 2021). The genetic background of Latinos was shaped by the fusion between waves of European migration, East Asian and African populations. Both European and African slaves came from different regions, as demonstrated in a well-prepared review by Gutiérrez-virgem et al (2023), providing a general overview of the population diversity observed in the main publications on genetic diversity of *NAT2* and ancestry in Latin America and in the world. The authors concluded that the genetic richness of contemporary Native Americans, together with varying degrees of admixture in their non-Native American populations, are important candidates for pharmacogenetic studies.

As in most South American countries, the process of admixture in Brazil was very diverse depending on the country region, which created a highly sub-structured population. Admixed populations has a peculiar evolutionary history, differing in parental sources, proportion and time of admixture. Furthermore, the admixture process produces variations at different levels: in ancestry between the populations, between individuals in the same population, and across the entire genome of the same admixed individual (Korunes and Goldberg, 2021).

Brazilian population had influence of three major ancestry groups: the Native Americans who already inhabited the region upon colonization, European, represented by the Portuguese colonizer, and Africans that were brought by Portuguese during the slave trade period. Later other people migrated to the country, including Spaniards, Italians, Germans, Syrians, Lebanese and Japanese, also contributing to the formation of the current population (Carvalho et al., 2008).

Brazil is a country of continental extension, and it is currently divided into five main geopolitical regions (North, Northeast, Central-West, Southeast and South) with diverse histories of colonization and settlement. The difficulty of exploring the country at the beginning of colonization made it possible for large urban centers to exist close to the coast and rural populations and native communities in the interior of the country, that although demographically less representative, still exist and maintain a strong Native American origin.

The genetics landscape can be observed in studies carried out in Brazil by Pereira et al (2012), Saloum de Neves Manta et al. (2013) and Resque et al (2016), the authors highlight the considerable amount of ethnic mixing that occurred throughout the country leading to Native Americans, Europeans and Africans ancestries to be incorporated into their gene pool during the last five centuries. More recently, contributions from other regions, such as East Asia and the Middle East were also seen.

In this context, the *NAT2* allelic distribution in populations characterized by a high degree of ethnic admixture, requires further investigation (Alves-Silva et al., 2000; Carvalho-Silva et al., 2001; Parra et al., 2003). The knowledge of the genetic diversity and haplotypic structure of the *NAT2* locus in ethnically admixed populations has important implications for understanding how *NAT2* genotype contributes to individual variation in drug response. In that context, the aim of this study was to determine the distribution of *NAT2* allelic and genotypic frequencies in populations living in five different geographical regions of Brazil. This allows a more global overview of *NAT2* genetic diversity and its inferred acetylation profile in the country.

## 2 Materials and methods

### 2.1 Subjects

After written informed consent, 964 unrelated individuals from five different geographical regions spanning ten states of Brazil were enrolled in this study. They comprised 240 active tuberculosis (TB) patients from the Federal University of Rio Grande do Sul (RS) in the South, 106 asymptomatic Healthcare Workers (HCWs) from Anuar Auad Infectious Disease Reference Hospital of the State Health Secretariat of Goiás State (GO) in the Midwest, and 618 leprosy patients from different units of the State Health Secretariat of Northeast, North and Southeast of Brazil, including the states of Acre (AC), Rondônia (RO) and Tocantins (TO), in the North (*n* = 190), Pernambuco (PE) and Rio Grande do Norte (RN), in the Northeast (*n* = 79), Espírito Santo (ES) and Rio de Janeiro (RJ), specifically the municipalities of Rio de Janeiro and Campos, in the Southeast (*n* = 339), and Paraná (PR) in the South (*n* = 10). The selection of patients in different regions was random, and the determination of genotype/haplotype was blinded.

The protocol was approved by the Ethics Committee of the Oswaldo Cruz Foundation (449/08) and a written informed consent prior to enrolment was obtained from each subject. The procedures followed were in accordance with the ethical standards of the responsible committee and with the Helsinki Declaration of 1975.

### 2.2 Sample collection and handling

A volume of 1 mL of venous blood was collected from each volunteer and stored at −20°C. Genomic DNA was isolated from 200 µL of frozen whole blood using the QIAamp^®^ DNA Blood Kit (Qiagen Inc., United States), according to the manufacturer’s specifications. After extraction, DNA samples were stored at −20°C.

### 2.3 *NAT2* genotyping

The *NAT2* genotyping was performed by sequencing of a 1,093 bp PCR-amplified DNA fragment spanning the entire coding exon of the gene, as previously described (Teixeira et al., 2007). Briefly, 100 ng of genomic DNA were added to a reaction mixture containing 100 ng of each primer (NAT2 EF and NAT2 ER; Teixeira et al., 2007), 0.2 mM of each dNTP, 3.5 mM MgCl2, 10% v/v glycerol, 10 mM Tris-HCl pH 9.0, 50 mM KCl, 0.1% v/v Triton X-100, and 1 U of Taq Gold DNA polymerase (Applied Biosystems, United States). Samples were subjected to a touchdown thermal cycling protocol for PCR amplification, comprising the following steps: initial denaturation for 5 min at 94°C; 20 cycles of 1 min at 94°C, 1 min at 67°C, then decreasing by 0.5°C per cycle, and 72°C for 1 min; 15 cycles of 1 min at 94°C, 1 min at 57°C and 1 min at 72°C; final extension for 5 min at 72°C. Evaluation of the amplified PCR product was carried out by electrophoresis on a 1% w/v agarose gel, followed by ethidium bromide (0.5 μg/mL) staining. The PCR products were purified with ChargeSwitch^®^ PCR Clean-UP Kit (Invitrogen, United States), according to the manufacturer’s specifications and subsequently used for direct Sanger sequencing. Because of the size of the amplified product (1,093 bp), and in order to read the entire coding sequence of *NAT2*, two internal oligonucleotides (NAT2 IF and NAT2 IR; Teixeira et al., 2007) were additionally used for sequencing of two overlapping fragments in opposite directions. Sequencing reactions were prepared using the ABI PRISM Big Dye Terminator v.3.1 Kit (Applied Biosystems) on an ABI 3730 automated sequencer (Applied Biosystems). Samples presenting new polymorphisms were re-amplified and re-sequenced for validation.

### 2.4 Sequence-based data analysis

Sequence data of each sample were analyzed for SNP identification after alignment with the sequence of the NAT2*4 allele (GenBank ID: AY331807), using SeqScape v.2.6 software (Applied BioSystems).

SNP frequencies were determined by chromosome counting, and deviations from Hardy-Weinberg equilibrium were tested by Arlequin v.3.0 (Excoffier et al., 2007). Haplotype reconstruction was performed using the Bayesian method implemented in PHASE v.2.1.1, which determines the most likely haplotype pair to obtain unambiguous genotypes (Stephens and Donnelly, 2003; Stephens and Scheet, 2005; Agúndez et al., 2008). This reconstruction is necessary, as commonly used genotyping methods for *NAT2*, including Sanger sequencing, cannot always discriminate between the two haplotypes of each genotype.

### 2.5 Statistical analysis

Statistical analysis was performed using the SPSS (Statistical Package for the Social Sciences) software version 17.0 for Windows. The chi-square test was used to determine whether there was a significant difference between the observed and expected frequencies, regarding the distribution of NAT2 variants in the analyzed cohort versus other populations. Statistical significance was assumed at *p* < 0.05.

## 3 Results

Genotyping of *NAT2* was performed for 964 samples (1928 chromosomes) from ten different states, across five geographical regions of Brazil: GO (Midwest), AC, RO and TO (North), RN and PE (Northeast), RJ and ES (Southeast), and PR and RS (South). Following amplification and sequencing of the entire intronless coding exon of *NAT2*, optimal quality sequences of 500–800 bp in length were produced. Their analysis allowed detection of *NAT2* SNPs, followed by the determination of alleles and genotypes, as well as the prediction of acetylator phenotypes.

### 3.1 Distribution of *NAT2* SNPs

The distribution and regional frequencies of detected SNPs are provided in Supplementary Table S1 for all individuals combined, as well as separately for each state and geographical region.

A total of 23 SNPs were identified in the 873 bp coding exon of *NAT2*. SNP c.33C>A, previously reported in Brazil (Spinasse et al., 2014), was found in four states across four of the five regions examined, and its frequency ranged from 0.7% to 5% (0.7% in TO, 2% in RJ, 2.9% in PE and 5% in PR).

**FIGURE 1:**
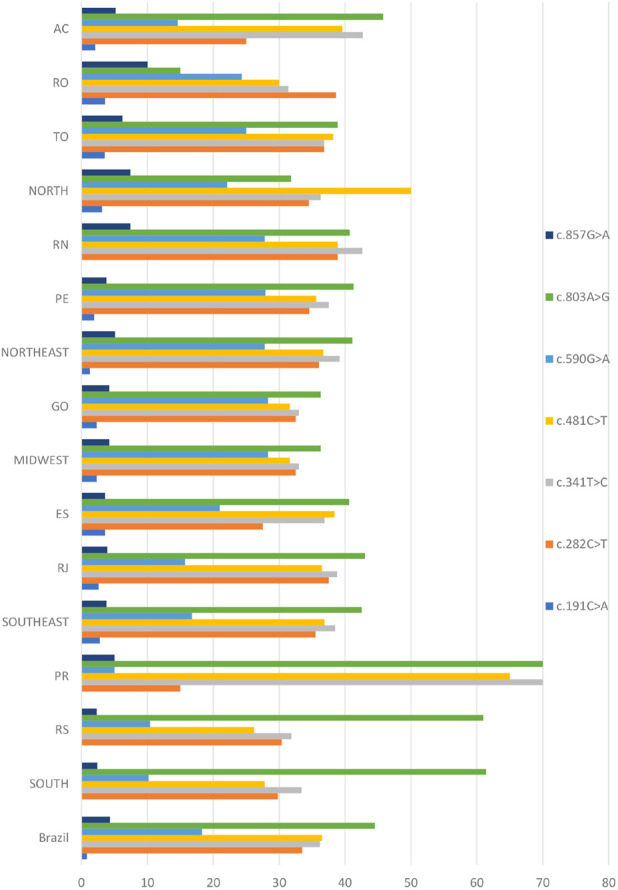
Distribution of SNPs found in the *NAT2* coding exon across the population of Brazil. The genetic analysis covered ten states in five regions of the country, including the municipality of Campos in the Rio de Janeiro area (for full names of states, see Section 2.1). Values are expressed as a percentage.

The seven most common SNPs reported in population genetic studies of *NAT2* variability were also encountered most frequently in our Brazilian cohort, which has an admixed African, European, and Native American ancestry (Figure 1 and Supplementary Table S1). SNP rs1208 (c.803A>G) was the most common overall, with a frequency range of 15%–70%, and a country average of 44.5%. SNP rs1799929 (c.481C>T) had a country average frequency of 36.5%, followed by rs1801280 (c.341T>C, frequency 36.2%), rs1041983 (c.282C>T, frequency 33.5%), rs1799930 (c.590G>A, frequency 18.3%), rs1799931 (c.857G>A, frequency 4.3%), and rs1801279 (c.191G>A, frequency 0.8%).

Comparison of SNP frequencies among the broader geographical regions demonstrated significant differences as observed in Table 1. There was no significant difference in frequency among those states where rs1801279 (c.191G>A) was found.

**TABLE 1:** Summary of statistically significant differences observed in the population frequencies of most common *NAT2* SNPs, among the various geographical regions and states of Brazil. *p* values are in bold.

c.282C>T (rs1041983)											
Region/State	South		Southeast			Midwest	North			Northeast	
	RS	PR	Campos	RJ	ES	GO	TO	RO	AC	PE	RN
AC							***p* = 0.05**				
c.341T>C (rs1801280)											
Region/State	South		Southeast			Midwest	North			Northeast	
	RS	PR	Campos	RJ	ES	GO	TO	RO	AC	PE	RN
RS		***p* = 0.025**									
c.481C>T (rs1799929)											
Region/State	South		Southeast			Midwest	North			Northeast	
	RS	PR	Campos	RJ	ES	GO	TO	RO	AC	PE	RN
RS		***p* = 0.004**									
South			***p* < 0.001**				***p* = 0.004**			***p* < 0.001**	
c.590G>A (rs1799930)											
Region/State	South		Southeast			Midwest	North			Northeast	
	RS	PR	Campos	RJ	ES	GO	TO	RO	AC	PE	RN
South			***p* < 0.001**			***p* = 0.002**	***p* < 0.001**			***p* < 0.001**	
Campos				***p =* 0.05**							
Southeast						***p* = 0.002**				***p* = 0.01**	
c.803A>G (rs1208)											
Region/State	South		Southeast			Midwest	North			Northeast	
	RS	PR	Campos	RJ	ES	GO	TO	RO	AC	PE	RN
RS		***p* < 0.001**				***p* < 0.001**				***p* < 0.001**	
South			***p* < 0.001**			***p* < 0.001**			***p* < 0.001**		
Southeast	***p* < 0.001**										
GO	***p* < 0.001**										
RO	***p* < 0.001**						***p* < 0.001**		***p* < 0.001**		
c.857G>A (rs1799931)											
Region/State	South		Southeast			Midwest	North			Northeast	
	RS	PR	Campos	RJ	ES	GO	TO	RO	AC	PE	RN
South			***p* = 0.004**				***p* < 0.001**				
GO	***p* = 0.018**										
Northeast	***p* = 0.011**										

### 3.2 Distribution of *NAT2* allelic, genotypic and phenotypic variability

The haplotype reconstruction from SNPs identified during sequencing analysis of the *NAT2* coding exon allowed a total of 44 allelic designations, nine of which were new, meaning they had not been previously described in the literature or on the *NAT* website (http://nat.mbg.duth.gr/). Database of arylamine N-acetyltransferase (2016) Eight individuals could not be phased. Therefore, the total number of samples for allelic and genotypic analyses was 956. The new haplotypes were assigned official symbols by the *NAT* Gene Nomenclature Committee and five of those contained the c.33C>A SNP previously described in Brazil (Spinasse et al., 2014). The frequencies of determined haplotypes are provided in Supplementary Table S2, while the new haplotypes are described in Table 2.

**TABLE 2:** New *NAT2* haplotypes determined for samples from different Brazilian regions^a^.

*NAT2*	*5AA	*5BA	*5BB	*5CA	*12Q	*12R	*12S	*13H	*28
SNP									
c.33C>A		•		•	•		•	•	
c.82C>T	•								
c.282C>T								•	
c.341T>C	•	•	•	•					
c.345C>T			•						
c.481C>T	•	•					•		
c.518A>G						•			
c.609G>T						•			
c.622T>C									•
c.803A>G		•	•	•	•	•	•		
Region	Frequency N^b^ ^(%)^								
North							1 ^(0.3)^		1 ^(0.3)^
Northeast		2 ^(1.3)^					1 ^(0.6)^		
Midwest			2 ^(0.9)^						
Southeast	1 ^(0.1)^	5 ^(0.7)^		1 ^(0.1)^	2 ^(0.3)^	1 ^(0.1)^		3 ^(0.4)^	
South	1 ^(0.2)^	1 ^(0.2)^							
Brazil	2 ^(0.1)^	8 ^(0.4)^	2 ^(0.1)^	1 ^(0.05)^	2 ^(0.1)^	1 ^(0.05)^	2 ^(0.1)^	3 ^(0.2)^	1 ^(0.05)^

^a^ New allelic symbols were assigned by the NAT Gene Nomenclature Committee (http://nat.mbg.duth.gr/), according to current consensus nomenclature (Vatsis et al., 1995; Hein et al., 2008; Boukouvala, 2018; Database of arylamine N-acetyltransferase, 2016).

^b^ N = 1,928 alleles analyzed.

**FIGURE 2:**
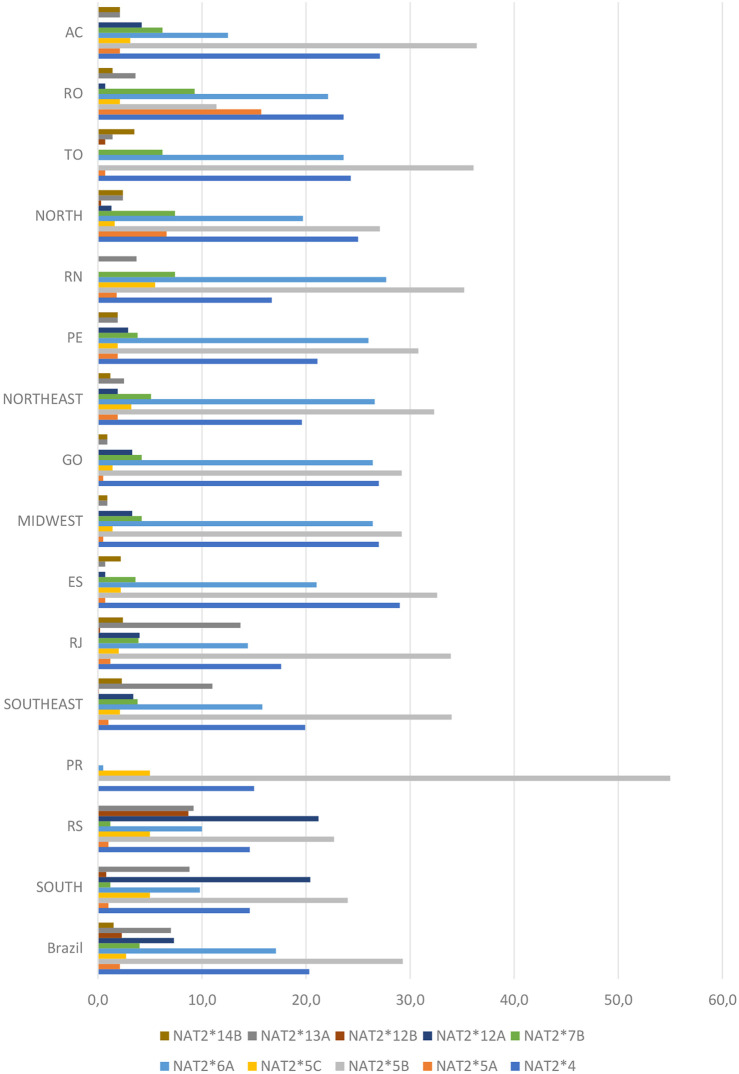
Distribution of *NAT2* alleles determined for the Brazilian population. The genetic analysis covered ten states in five regions of the country, including the municipality of Campos in the Rio de Janeiro area (for full names of states, see Section 2.1). Values are expressed as a percentage.

Ten alleles demonstrated frequencies higher than 1% in at least one state and those contained various combinations of the seven most common *NAT2* SNPs mentioned above. Among them, four rapid alleles (NAT2*4, NAT2*12A, NAT2*12B and NAT2*13A) and six slow alleles (NAT2*5A, NAT2*5B, NAT2*5C, NAT2*6A, NAT2*7B and NAT2*14B) were observed (Figure 2 and Table 3). The comparison of allele frequencies among geographical regions demonstrated significant differences, as presented in Table 4.

**TABLE 3:** *NAT2* haplotypes determined with frequencies above 1% in samples from different Brazilian regions. *p* values are in bold.

	NAT2*4	NAT2*5A	NAT2*5B	NAT2*5C	NAT2*6A	NAT2*7B	NAT2*12A	NAT2*12B	NAT2*13A	NAT2*14B	SNP effect
SNP											
c.191G>A (rs1801279)	G	G	G	G	G	G	G	G	G	**A**	Non-synonymous^a^
c.282C>T (rs1041983)	C	C	C	C	**T**	**T**	C	**T**	**T**	**T**	Synonymous^b^
c.341T>C (rs1801280)	T	**C**	**C**	**C**	T	T	T	T	T	T	Non-synonymous^a^
c.481C>T (rs1799929)	C	**T**	**T**	C	C	C	C	C	C	C	Synonymous^b^
c.590G>A (rs1799930)	G	G	G	G	**A**	G	G	G	G	G	Non-synonymous^a^
c.803A>G (rs1208)	A	A	**G**	**G**	A	A	**G**	**G**	A	A	Non-synonymous^b^
c.857G>A (rs1799931)	G	G	G	G	G	**A**	G	G	G	G	Non-synonymous^a^
Phenotype	Rapid	Slow	Slow	Slow	Slow	Slow	Rapid	Rapid	Rapid	Slow	
State/Region	Frequency (%)										N^c^
North	**25.1**	**6.6**	**27.2**	**1.6**	**19.6**	**7.4**	**1.3**	**0.3**	**2.4**	**2.4**	**378**
RO	24.2	15.7	11.4	2.1	21.4	9.3	0.7	0	3.6	1.4	140
AC	27	2.1	36.4	3.1	12.5	6.2	4.2	0	2.1	2.1	96
TO	24.6	0.7	36.6	0	22.5	6.3	0	0.7	1.4	3.5	142
Northeast	**19.8**	**1.9**	**32**	**3.2**	**26.9**	**5.1**	**1.9**	**0**	**2.6**	**1.3**	**156**
PE	21.5	1.9	30.4	2	26.5	3.9	2.9	0	1.9	1.9	102
RN	16.6	1.8	35.2	5.5	27.8	7.4	0	0	3.7	0	54
Midwest	**26.8**	**0.5**	**29.2**	**1.4**	**26.4**	**4.2**	**3.3**	**0**	**0.9**	**0.9**	**212**
GO	26.8	0.5	29.2	1.4	26.4	4.2	3.3	0	0.9	0.9	212
Southeast	**20**	**1**	**33.8**	**2.1**	**16**	**3.9**	**3.4**	**0.1**	**11.2**	**2.4**	**670**
RJ^d^	17.6	1.2	33.8	2.1	18.7	3.9	4	0.2	13.9	2.4	532
ES	28.9	0.7	34	2.2	21	3.6	0.7	0	0.7	2.2	138
South	**14.7**	**1**	**23.8**	**5**	**9.9**	**1.2**	**21**	**8.4**	**8.8**	**0**	**496**
PR	16.6	0	61.1	5.5	5.5	0	0	0	0	0	18
RS	14.6	1	22.4	5	10	1.3	21.7	8.7	9.2	0	478
Total	**20.4**	**2.1**	**29.3**	**2.8**	**17.1**	**4**	**7.4**	**2.3**	**7**	**1.5**	**1,912**

^a^ Phenotypically non-conservative (slow).

^b^ Phenotypically conservative (no effect).

^c^ Number of alleles.

^d^ Samples from the municipalities of Rio de Janeiro and Campos combined.

**TABLE 4:** Summary of statistically significant differences observed in the population frequencies of most common *NAT2* alleles/haplotypes, among the various geographical regions and states of Brazil. *p* values are in bold.

NAT2*4											
Region/State	South		Southeast			Midwest	North			Northeast	
	RS	PR	Campos	RJ	ES	GO	TO	RO	AC	PE	RN
RS		***p* < 0.001**									
South			***p* < 0.001**			***p* < 0.001**	***p* < 0.001**			***p* < 0.001**	
RJ					***p* = 0.008**						
NAT2*5A											
Region/State	South		Southeast			Midwest	North			Northeast	
	RS	PR	Campos	RJ	ES	GO	RO	TO	AC	PE	RN
RO	***p* < 0.001**		***p* < 0.001**			***p* < 0.001**		***p* < 0.001**		***p* < 0.001**	
NAT2*5B											
Region/State	South		Southeast			Midwest	North			Northeast	
	RS	PR	Campos	RJ	ES	GO	RO	TO	AC	PE	RN
RS		***p* = 0.001**	***p* < 0.001**				***p* = 0.004**	***p* < 0.001**	***p* < 0.001**	***p* = 0.002**	
PR						***p* = 0.025**				***p* = 0.045**	
Southeast								***p* < 0.001**			
RO								***p* < 0.001**		***p* < 0.001**	
NAT2*6A											
Region/State	South		Southeast			Midwest	North			Northeast	
	RS	PR	Campos	RJ	ES	GO	TO	RO	AC	PE	RN
South			***p =* 0.004**			***p* < 0.001**				***p* < 0.001**	
Southeast						***p* < 0.001**				***p* = 0.001**	
AC						***p =* 0.008**	***p =* 0.045**			***p =* 0.008**	
Northeast							***p* < 0.001**				
NAT2*7B											
Region/State	South		Southeast			Midwest	North			Northeast	
	RS	PR	Campos	RJ	ES	GO	TO	RO	AC	PE	RN
South						***p* = 0.014**	***p* = 0.001**			***p* = 0.004**	
RJ	***p* = 0.008**				***p* = 0.025**						
ES						***p* = 0.014**	***p* = 0.001**			***p* = 0.008**	
NAT2*12A											
Region/State	South		Southeast			Midwest	North			Northeast	
	RS	PR	Campos	RJ	ES	GO	TO	RO	AC	PE	RN
Campos				***p* = 0.014**	***p* = 0.005**		***p* = 0.005**			***p* = 0.025**	
AC							***p* = 0.014**				
NAT2*12B											
Region/State	South		Southeast			Midwest	North			Northeast	
	RS	PR	Campos	RJ	ES	GO	TO	RO	AC	PE	RN
South			***p* < 0.001**			***p* < 0.001**	***p* < 0.001**			***p* < 0.001**	
NAT2*13A											
Region/State	South		Southeast			Midwest	North			Northeast	
	RS	PR	Campos	RJ	ES	GO	TO	RO	AC	PE	RN
RJ	***p* < 0.001**		***p* = 0.001**		***p* = 0.001**	***p* < 0.001**	***p* < 0.001**			***p* < 0.001**	
NAT2*14B											
Region/State	South		Southeast			Midwest	North			Northeast	
	RS	PR	Campos	RJ	ES	GO	TO	RO	AC	PE	RN
South			***p* < 0.001**			***p* = 0.045**	***p* < 0.001**			***p* = 0.014**	

Based on their SNP composition, it was possible to deduce the acetylation phenotype for the majority of the *NAT2* haplotypes identified in the present study. The most common rapid alleles were NAT2*4 (reference, 20.4%), *12A (7.4%), *13A (7%), *12B (2.3%), *12C (0.7%) and *11A (0.2%). The most common slow alleles were NAT2*5B (29.3%) and NAT2* 6A (17.1%), in all states except RO. Those two slow alleles, together accounted for 46.4% of haplotypes in the population studied. The six most frequent defective alleles accounted for 56.8% of the *NAT2* haplotypes found in the Brazilian population.

In total, 116 different allelic diplotypes (genotypes) were identified in the populations studied from the five geographical regions of Brazil (Supplementary Table S3). Regarding the inferred acetylator phenotype, the overall population frequencies observed were 17.8% for rapid acetylators, 37.4% for slow acetylators, and 37.8% for intermediate acetylators (Supplementary Table S4). It was not possible to determine the phenotype in 6.9% of the samples.

The 21 genotypes, which demonstrate a population frequency at least 1% in Brazilian samples, are described in Table 5. Of those, seven (32.9%) genotypes conferred the slow acetylator phenotype, eight (30.6%) conferred the intermediate acetylator phenotype, and six (16.3%) the rapid acetylator phenotype. All observed genotype frequencies were in good agreement with the Hardy-Weinberg law.

**TABLE 5:** Distribution of determined *NAT2* genotypes with frequencies at least 1% in Brazilian samples.

*NAT2* genotypes	Number of subjects (% frequency)	Brazil				
		South, % (*N* = 248)^a^	Southeast, % (*N* = 335)^a^	Midwest, % (*N* = 106)^a^	Northeast, % (*N* = 78)^a^	North, % (*N* = 189)^a^
Rapid acetylator phenotype						
*4/*4	55 (5.7%)	6.4	3.9	8.5	6.4	6.3
*4/*13A	41 (4.3%)	9.6	4.2	0	0	1.6
*12A/*12A	23 (2.4%)	8.8	0	0.9	0	0
*13A/*13A	16 (1.6%)	3.2	2.4	0	0	0
*12A/*12B	12 (1.2%)	4.8	0	0	0	0
*4/*12A	11 (1.1%)	0.41	1.8	1.9	0	1.0
Slow acetylator phenotype						
*5B/*5B	109 (11.4%)	10.5	12.2	11.3	9.0	12.1
*5B/*6A	87 (9.1%)	2.4	11.6	11.3	18.0	8.4
*6A/*6A	54 (5.6%)	7.2	2.9	8.5	7.6	5.8
*5B/*7B	25 (2.6%)	0.8	3.0	0.9	5.1	4.2
*6A/*7B	17 (1.8%)	0	1.2	3.8	3.8	3.2
*5A/*6A	12 (1.2%)	0.8	0.6	0.94	2.5	2.6
*5B/*5C	12 (1.2%)	0	2.4	0.9	1.3	1.0
Intermediate acetylator phenotype						
*4/*5B	96 (10.0%)	2.4	13.1	16.0	12.8	10.0
*4/*6A	68 (7.1%)	1.2	7.2	14.1	7.7	10.6
*5B/*12A	37 (3.9%)	11.3	1.8	0.9	0	1.0
*5B/*13A	30 (3.1%)	0.4	6.8	0.9	3.8	1.0
*5B/*12B	24 (2.5%)	8.8	0.3	0	0	0.5
*4/*5A	13 (1.4%)	0	0.6	0	1.3	5.3
*4/*7B	13 (1.4%)	0.4	0.9	1.9	1.3	3.2
*5C/*12A	12 (1.2%)	4.3	0.3	0	0	0

^a^ *N* = number of samples.

**FIGURE 3:**
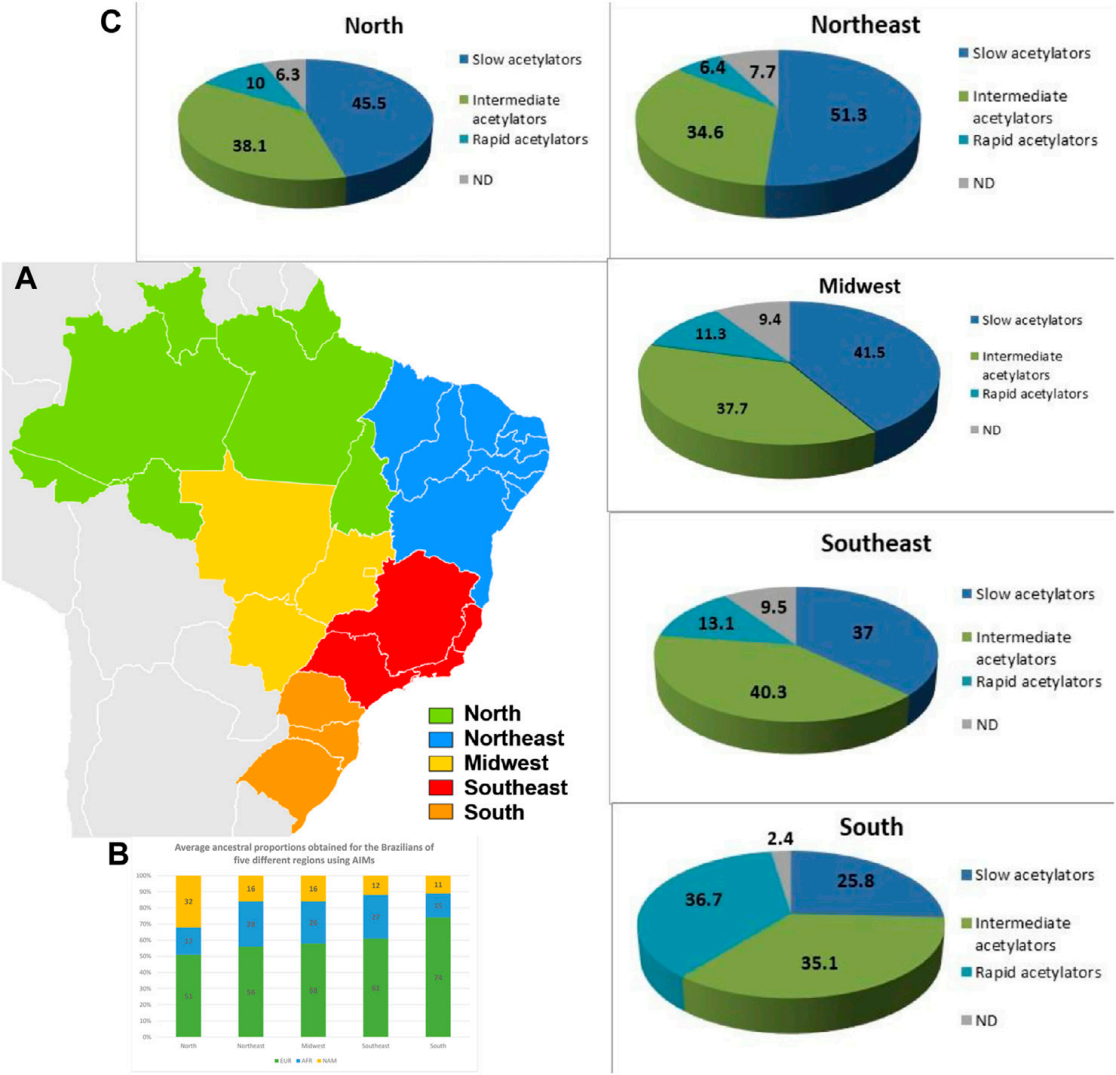
Distribution of ethnic and acetylation profiles along Brazilian territory. **(A–C)** Distribution of *NAT2* phenotypes inferred for the Brazilian population by region. Values are expressed as a percentage. ND, not determined.

There were significant differences in the frequencies of common genotypes between states and regions. Genotype NAT2*4/*4 showed significant difference (*p* < 0.001) between the RJ and ES states in the Southeast, as well as between RJ and all other regions, i.e., the South (*p* = 0.014), the Northeast (*p* = 0.045), the Midwest (*p* = 0.002) and the North (*p* = 0.014). Genotype NAT2*12A/*12A was found only in RS (9.2%) and GO (0.9%) states, and its frequency varied significantly in the South relative to other regions (*p* < 0.001). Genotypes NAT2*12A/*12B and NAT2*12A/*12C were found only in the RS state in frequencies of 5% and 1.7%, respectively, and varied relative to other regions (*p* < 0.001). Genotype NAT2*13A/*13A was also found in RS (3.3%) and RJ (3%) states, and its frequency varied significantly relative to other regions (*p* < 0.001). After the genotyping was completed (Supplementary Table S5), it was possible to map the distribution of different acetylator phenotypes in the five analysed regions of Brazil (Figure 3). The South presented significant difference in the acetylator profile, compared with all other regions (*p* < 0.001). Similarly, a difference was also seen between the Southeast and Northeast region (*p* = 0.014).

### 3.3 Interethnic diversity

To gain further insight into how the Brazilian population compares with others, we also surveyed studies recording *NAT2* genetic variability in other geographical areas. An overview of representative studies reporting on the frequencies of common *NAT2* haplotypic groups across global populations is presented in Table 6. As previously observed, the relative frequencies vary considerably among different ethnic backgrounds. Moreover, although Brazilians presented all common alleles reported for different continental groups, the corresponding population frequencies were often statistically different (Table 6).

**TABLE 6:** Frequencies of *NAT2* allelic groups compared between populations worldwide.

Population (N)	Geographical location	Reference	*NAT2* haplotypes, % (*p*-value)						
			*4	*5	*6	*7	*12	*13	*14
America									
**Brazilian (964)**	**Brazil**	This study	20.4^R^	36.0^R^	18.2^R^	4.2^R^	11.3^R^	7.4^R^	2^R^
Ngawbe (105)	Panama	Jorge-Nebert et al. (2002)	72.4 ^(*p* ˂ 0.001)^	2.4 ^(*p* ˂ 0.001)^	0 ^(*p* ˂ 0.001)^	23.3 ^(*p* ˂ 0.001)^	0 ^(*p* ˂ 0.001)^	1.9 ^(*p* = 0.004)^	ND
Embera (136)	Panama	Jorge-Nebert et al. (2002)	61 ^(*p* ˂ 0.001)^	9.9 ^(*p* ˂ 0.001)^	3.7 ^(*p* ˂ 0.001)^	22.8 ^(*p* ˂ 0.001)^	0 ^(*p* ˂ 0.001)^	2.6 ^(*p* = 0.004)^	ND
Africa									
Tswana (101)	South Africa	Loktionov et al. (2002)	13.4 ^(*p* = 0.025)^	32.2	20	0 ^(*p* = 0.004)^	20.8 ^(*p* ˂ 0.001)^	6.4	8.4 ^(*p* ˂ 0.001)^
San (40)	Zimbabwe	Matimba et al. (2009)	23.7	20 ^(*p* = 0.004)^	20	1.3	23.8 ^(*p* ˂ 0.001)^	2.5	8.8 ^(*p* ˂ 0.001)^
Mandenka (97)	Senegal	Sabbagh et al. (2008)	9.3 ^(*p ˂* 0.001)^	36.1	17	6.7	15.5	5.2	10.3 ^(*p* ˂ 0.001)^
Sudanese (127)	Sudan	Al-Yahyaee et al. (2007)	8.7 ^(*p* ˂ 0.001)^	47.2 ^(*p* ˂ 0.001)^	28.7 ^(*p* ˂ 0.001)^	3.1	8.3	0.8 ^(*p* ˂ 0.001)^	3.1
Europe									
Spanish (1,312)	Spain	Agúndez et al. (2008)	22.2	45.7 ^(*p* ˂ 0.001)^	26.7 ^(*p* ˂ 0.001)^	1.2 ^(*p* ˂ 0.001)^	2.6 ^(*p* ˂ 0.001)^	0.3 ^(*p* ˂ 0.001)^	1.4
French (60)	France	Deloménie et al. (1996)	18.3	51.7 ^(*p* ˂ 0.001)^	25	0.8	0 ^(*p* ˂ 0.001)^	4.2	0
German (844)	Germany	Cascorbi et al. (1995)	22.7	46.5 ^(*p* ˂ 0.001)^	27.8 ^(*p* ˂ 0.001)^	1.3 ^(*p* ˂ 0.001)^	ND	1.5 ^(*p* ˂ 0.001)^	0.1 ^(*p* ˂ 0.001)^
UK Caucasian (112)	United Kingdom	Loktionov et al. (2002)	19.6	52.7 ^(*p* ˂ 0.001)^	24.6 ^(*p* = 0.025)^	2.2	0.4 ^(*p* ˂ 0.001)^	0.4 ^(*p* ˂ 0.001)^	0.9
US Caucasian (387)	United States	Deitz et al. (2000)	24.2 ^(*p* = 0.045)^	45.9 ^(*p* ˂ 0.001)^	26.6 ^(*p* ˂ 0.001)^	1.9 ^(*p* = 0.004)^	0.4 ^(*p* ˂ 0.001)^	0.9 ^(*p* ˂ 0.001)^	0.1 ^(*p* ˂ 0.001)^
Polish (248)	Poland	Mrozikiewicz et al. (1996)	22	44.4 ^(*p* ˂ 0.001)^	30 ^(*p* ˂ 0.001)^	3.4	0.2 ^(*p* ˂ 0.001)^	0 ^(*p* ˂ 0.001)^	0 ^(*p* = 0.002)^
Russian (364)	Russia	Belogubova et al. (2005)	23.5	45.6 ^(*p* ˂ 0.001)^	27.2 ^(*p* ˂ 0.001)^	3.2	0.5 ^(*p* ˂ 0.001)^	0^(*p* ˂ 0.001)^	0 ^(*p* ˂ 0.001)^
Asia									
Thai (44)	Thailand	Patin et al. (2006b)	29.5 ^(*p* = 0.045)^	11.4 ^(*p* ˂ 0.001)^	38.6 ^(*p* ˂ 0.001)^	19.3 ^(*p* ˂ 0.001)^	0 ^(*p* = 0.001)^	1.1 ^(*p* = 0.045)^	0
Han Chinese (212)	China	Song et al. (2009)	59.2 ^(*p* ˂ 0.001)^	4 ^(*p* ˂ 0.001)^	20.8	14.9 ^(*p* ˂ 0.001)^	0.7 ^(*p* ˂ 0.001)^	0.5 ^(*p* ˂ 0.001)^	0 ^(*p* = 0.004)^
Japanese (200)	Japan	Machida et al. (2005)	69.5 ^(*p* ˂ 0.001)^	0.5 ^(*p* ˂ 0.001)^	19.8	8.8 ^(*p* ˂ 0.001)^	ND	1.3 ^(*p* ˂ 0.001)^	ND
Korean (1,000)	Korea	Lee et al. (2002)	66.1 ^(*p* ˂ 0.001)^	1.6 ^(*p* ˂ 0.001)^	20.1	11.5 ^(*p* ˂ 0.001)^	0.8 ^(*p* ˂ 0.001)^	0.1 ^(*p* ˂ 0.001)^	0 ^(*p* ˂ 0.001)^

R, Reference group; ND, not determined. See also Mortensen et al. (2011a), Sabbagh et al. (2011), Patillon et al. (2014), and Podgorná et al. (2015).

## 4 Discussion

In the present study, we have analyzed the sequence of *NAT2* coding exon in 1928 chromosomes sampled from Brazilians in five different geographical regions of the country. The activity of the NAT2 enzyme encoded by this highly polymorphic gene has important implications for health risk assessment of environmental agents, given the critical importance of this enzyme in activating some types of aromatic amines and hydrazines, while detoxifying others. Consequently, it has been established that variation in the acetylator phenotype may lead to an increased risk for drug adverse reactions or lack of therapeutic efficacy (Teixeira et al., 2011; Azuma et al., 2012; Gupta et al., 2013; Spinasse et al., 2014; Adithan and Subathra, 2016), while it may also be linked to a variety of complex human disorders, such as several malignancies, atopic diseases, diabetes, Parkinson’s disease and others (Hein, 2000; Butcher et al., 2002; McDonagh et al., 2014; Liang et al., 2015; Chang et al., 2016; Luan et al., 2017).

Inter- and intra-ethnic variability of *NAT2* genotype and phenotype has been described in populations around the world and is attributed to a small number of common *NAT2* SNPs associated with low acetylation capacity. The distribution of those alleles has been extensively studied in Europeans, Africans, and Asians; however, their frequency varies considerably among populations and sub-populations around the world, stressing the need to investigate *NAT2* polymorphism in as many populations as possible, particularly those underrepresented in earlier genetic studies. Here, we described the distribution and frequency of 23 *NAT2* SNPs, including the seven most encountered ones worldwide. The presence of SNPs in significantly variable frequencies, across ten states and five broad geographical regions of the country, allowed a more comprehensive overview of the haplotypic, genotypic and phenotypic *NAT2* variability pattern in the highly admixed Brazilian population.

The Brazilian territory, of great continental extension, was firstly occupied by North Americans indigenous, as reported by Campelo-dos-Santos et al. (2022), who presented substantial genomic evidence of ancient migratory events along the coast Atlantic of South America.

The ethnic admixture process in Brazil is very diverse depending on the region (Saloum de Neves Manta et al,. 2013)—Figure 3). Its occupation began on the coast with the arrival of the Portuguese at the beginning of the 16th century, the Brazilian territory previously occupied by indigenous North Americans came to be influenced by three main groups: the Native Americans, who already inhabited the region, the Portuguese, who arrived in the mid-1500s, and the Africans who were brought by the Portuguese during the period of slavery. Consequently, the progress of colonization was highly diverse in the different regions as reflected in the genetic structure of the current Brazilian population (Alves-Silva et al., 2000; Carvalho et al., 2008; Resque et al., 2010; Resque et al., 2016) which was also influenced by the immigration of other populations such as Spaniards, Italians, Germans, Syrians, Lebanese, Japanese, Koreans and Chinese.

This panel of heterogeneity is well shown by Escher et al. (2022), where tri-and-tetra-hybrid models were analyzed and when comparing these models in other mixed populations in America for which there are no historical records of large flows migrants from the EAS (except Peru), unlike the Brazilian population, no significant differences were observed in the population means of the components between the mixed models.

In studies carried out in Brazil and very well documented by Pereira R et al (2012), Saloum de Neves Manta et al. (2013), the ethnic admixture throughout the country is clearly shown by using a panel of 46 Indels Ancestry Informative Markers (AIMs) to characterize and compare the genetic composition of more than 1,300 individuals from 14 populations across the five geopolitical regions (Figure 3).

Several *NAT2* SNPs with known effects on the acetylator phenotype have been observed previously at high frequencies in specific ethnic groups. SNP c.191G>A (signature for the slow NAT2*14 allelic group) is mainly found in African populations and is absent in European descendants (Bell et al., 1993; Deloménie et al., 1996; Cavaco et al., 2003). In our cohort, this SNP was found in four Brazilian regions, in a relatively low frequency (1.3%–3.1%) which was, however, higher than the frequency seen in the Europeans and more indicative of African origin. On the other hand, SNP c.857G>A (signature for the slow NAT2*7 allelic group) is predominant in East Asian populations (e.g., in frequencies of 30%–33% in the Korean and Chinese) but is much rarer (1%–5%) in European descendants (Lin et al., 1993). Jorge-Nebert et al. (2002) also showed this SNP to be present in Native American populations. In our study, the c.857G>A SNP was found in samples from all analyzed states, with frequencies varying from 2.3% to 10%, and RO (10%), RN (7.4%), TO (6.2%) and AC (5.2%) identified as the states with highest prevalence of this SNP. These results are in accordance with the findings of Jorge-Nebert and are not surprising given the greater proportion of indigenous population in that area. In addition, they also highlight the extensive admixture in the genetic constitution of Brazilians.

Distribution of *NAT2* SNPs c.341T>C (signature for the slow NAT2*5 allelic group), c.481C>T and c.803A>G has also been correlated with ethnicity. Those SNPs are usually prevalent in European descendants and Africans. In contrast, SNP c.590G>A (signature for the slow NAT2*6 allelic group) shows a rather homogenous frequency distribution across global populations (Cascorbi et al., 1995; Agúndez et al., 1996; Deloménie et al., 1996; Jorge-Nebert et al., 2002; Cavaco et al., 2003; Sabbagh et al., 2011; Patillon et al., 2014; Sabbagh et al., 2018). In our study, SNPs c.341T>C (31.4%–70%) and c.803A>G (15%–70%) were the most prevalent ones, followed by SNPs c.481C>T (26.2%–65%) and c.590G>A (5%–28.3%). The distribution was indicative of the strong European influence on the genetic profile of *NAT2* in Brazilians. It is worth noting that, for the Southern state of Paraná, only ten individuals were genotyped, leading to considerable deviation in the frequencies observed for this particular sub-population. Increasing the sample size, especially for this state, would be necessary to confirm this apparent differentiation. In RS (South) and RN (Northeast) states, only six of the seven most frequent SNPs were detected. SNP c.191G>A was not found in both states. Considering all identified SNPs in relation to their predicted effect on *NAT2* enzymatic activity, it can be concluded that the frequency of SNPs associated with low enzymatic activity was higher in RN (77%) than in RS (44.6%).

The c.282C>T is widely distributed across worldwide populations (Sabbagh et al., 2011) and the same pattern was observed among different Brazilian states and regions. Significant difference was observed only when its frequency in AC (25%) was compared with other Northern states.

The variability distribution of *NAT2* in the Brazilian population corroborates previous observations of our group. Teixeira et al. (2007) compared the allelic diversity of *NAT2* between residents of RJ (Southeast) and GO (Midwest) states, demonstrating the heterogeneity of the Brazilian population. The authors attributed their findings to the influence of different ethnic backgrounds introduced into the Brazilian population during colonization. Although that early study was performed in only two states (RJ and GO), it demonstrated the importance of involving additional regions of Brazil. In a subsequent study (Possuelo et al., 2008), the pattern of *NAT2* genetic variability was additionally investigated in the RS (South) state. More importantly, that study confirmed the association between the *NAT2* slow acetylator phenotype and drug-induced adverse reactions in TB patients treated with therapeutic schemes including INH. The results supported those of studies previously performed with ethnically less diverse populations. It also characterized *NAT2* variability in different regions of Brazil, including the different ethnicities represented in the gene pool of Brazilians, a population with admixed European, African and Native American roots.

According to the molecular genetics of *NAT2*, where alleles are characterized by the combination of one to six different SNPs, haplotype characterization is fundamental for the prediction of the acetylation phenotype. After haplotype reconstruction, we identified 44 alleles/haplotypes, distributed variably between states. The most frequent allele was NAT2*5B (29.3%), followed by NAT2*4 (20.4%) and NAT2*6A (17.1%), in line with the findings of Teixeira et al. (2007) for Brazil and the findings of Sabbagh et al. (2011) who evaluated the genetic diversity of NAT2 in 99 population samples. Haplotypes with non-determined phenotypes were represented with a frequency of 3.5% in the current population sample.

Among the functional alleles, NAT2*4 was the most frequent in all states, except for RS (South) where the rapid acetylation phenotype was attributed mainly to the NAT2*13A and *12B alleles. In our population, the reference NAT2*4 allele showed frequencies like those found in European populations and was significantly more prevalent compared with central African (*p* < 0.001) and South African (*p* = 0.025) populations. The distribution in the whole sample varied for functional alleles other than NAT2*4, i.e., NAT2*12A (7.4%), NAT2*13A (7%), NAT*12B (2.3%) and NAT2*12C (0.7%). In fact, the distribution of those alleles showed some noteworthy features across different sub-populations. For example, allele NAT2*13A had a low frequency in most of states, except for RS (9.2%) and RJ (13.9%, for the municipalities of Rio de Janeiro and Campos combined). Moreover, among the two RJ municipalities, the frequency of NAT2*13A was almost four times higher in Rio de Janeiro (16.5%) versus Campos (4.4%). The NAT2*12A allele, which had a low frequency in the majority of the states, was encountered in higher frequencies in Campos (7.9%) and RS (21.7%). The NAT2*12B allele was found only in Rio de Janeiro municipality (0.2%), TO (0.7%) and RS (8.7%), while the *12C allele was found only in RO (0.7%), TO (0.7%), ES (2.2%) and RS (1.9%). Interestingly, those four alleles were not found at all in RN or in PR.

Haplotypic groups NAT2*5 and NAT2*6 were the most frequent defective alleles in the Brazilian population. NAT2*5B showed relatively lower frequencies in RO (11.4%) and RS (22.4%); for all other states, the allele had higher frequencies (∼30% or higher). Moreover, the high frequency (61%) of NAT2*5B in PR was found to be significantly different when compared to the Midwest (*p* = 0.025) and the Northeast (*p* = 0.045) regions (∼30%). The allelic frequency of NAT2*5A was highest in RO (15.7%), differing significantly from AC and TO (also in the North), and all other regions (*p* < 0.001). Finally, the second most frequent defective allele, NAT2*6A, varied significantly when the Midwest and Northeast (∼26.5%) were compared with the Northern AC state (12.5%), as well as with the South (9.9%) and Southeast (16%) regions. The Asian allele NAT2*7B showed its highest frequencies in the North (7.4%) and Northeast (5.1%) regions. Significant differences were observed between the South (1.2%) and North (7.4%), Northeast (5.1%) and Midwest (4.2%) regions. Finally, the NAT2*14B allele demonstrated significant difference in frequency between the South (undetermined frequency) and all other regions (∼1% or higher).

The low-activity allelic group NAT2*5 has been reported as the most frequent in all European and African populations, and this was the case for our population too. Interestingly, although the frequency of this allelic group was significantly lower in Brazilians, compared with populations of European or African descent (*p* < 0.005), it was nevertheless significantly higher than in the Japanese, Chinese, Korean, Thai and Native American populations (*p* < 0.001). The NAT2*6 was the second most frequent low-activity allelic group in our population, and it was well-distributed in our cohort. The frequency of this allelic group in the Brazilian population was significantly lower than in the European (*p* < 0.001), Thai (*p* < 0.001) and Sudanese (*p* < 0.001) population. The NAT2*7 allelic group, also associated with low NAT2 activity, is mainly found in Asia and Central America, and its frequency was significantly lower in our Brazilian population than in the Japanese, Thai, Chinese, Korean and Native American (*p* < 0.001), but was higher than in some Caucasian populations. Finally, the NAT2*14 allelic group, which is more prevalent in Sub-Saharan Africa with a frequency above 6%, was also identified in our population in a lower frequency of about 2%. These findings clearly demonstrate that the contribution of different ethnic groups in Brazilian genetic background has an impact in the distribution of *NAT2* alleles throughout the country’s territory.

From the subsequent determination of *NAT2* allelic diplotypes (genotypes), it was possible to infer the acetylator phenotypes for individuals across the different states of Brazil. We observed that populations in the Northeast (RN and PE), North (RO, AC, and TO) and Midwest (GO) displayed a higher prevalence of slow acetylators (51.3%, 45.5% and 41.5%, respectively). On the other hand, in the Southeast, the most frequent phenotype was the intermediate acetylator (40.3%). Interestingly, the Southern state of RS presented the highest frequency of rapid acetylators (38%), with a significant difference compared to other states of Brazil (*p* < 0.0001). On the other hand, there was no significant difference in the phenotypic profile between the North and Northeast, suggesting homogeneity between the two regions. However, both regions showed a significant difference in the observed phenotypic pattern, when compared with the Southeast region (*p* = 0.04). More remarkably, when compared with the South region, all other regions of the country demonstrated significant difference (*p* < 0.0001), as the frequency of rapid acetylators in the South was considerably higher. Our findings demonstrate a gradual shift from higher prevalence of the slow acetylator phenotype in the North and more central parts of the country, to a higher prevalence of intermediate acetylators in the Southeast and of rapid acetylators in the South.

In conclusion, our results can be summarized in three fundamental observations: first, they reveal a significant diversity of the *NAT2* gene concerning SNPs, alleles and genotypes in the Brazilian population; second, the distribution of these diverse alleles varies among subpopulations across the country and third, these distribution patterns have an impact on the observed acetylation status, in line with previous literature (Meyer and Zanger, 1997; Jorge-Nebert et al., 2002; Dandara et al., 2003; Teixeira et al., 2007; Sabbagh et al., 2011). Given the clinical data showing unequivocal association between specific *NAT2* genotypes and susceptibility to some unfavorable outcomes of certain drugs, this study importantly contributes to the reconstruction of a more accurate phenotypic picture of the NAT2 acetylator profiles in different geographic regions of Brazil. We understand that our findings can be useful in planning therapeutic regimens containing drugs metabolized by NAT2 in these regions of Brazil.

## Data Availability

The original contributions presented in the study are included in the article/Supplementary Materials, further inquiries can be directed to the corresponding author.
